# Investigation of the Uptake and Transport of Aspirin Eugenol Ester in the Caco-2 Cell Model

**DOI:** 10.3389/fphar.2022.887598

**Published:** 2022-05-04

**Authors:** Qi Tao, Zhe Qin, Xi-Wang Liu, Zhen-Dong Zhang, Shi-Hong Li, Li-Xia Bai, Jian-Yong Li, Ya-Jun Yang

**Affiliations:** Key Lab of New Animal Drug Projectof Gansu Province, Key Lab of Veterinary Pharmaceutical Development of Ministry of Agriculture and Rural Affairs, Lanzhou Institute of Husbandry and Pharmaceutical Sciences of CAAS, Lanzhou, China

**Keywords:** aspirin eugenol ester, salicylic acid, Caco-2 cells, uptake, transport

## Abstract

**Background:** Aspirin eugenol ester (AEE) is a novel medicinal compound synthesized by esterification of aspirin with eugenol using the prodrug principle. AEE has the pharmacological activities of being anti-inflammatory, antipyretic, analgesic, anti-cardiovascular diseases, and anti-oxidative stress However, its oral bioavailability is poor, and its intestinal absorption and transport characteristics are still unknown.

**Objective:** The purpose of this study was to investigate the uptake and transport mechanisms of AEE in Caco-2 cells.

**Methods:** The effects of time, concentration, and temperature on the transport and uptake of AEE were studied.

**Results:** The results showed that a higher concentration of salicylic acid (SA) was detected in the supernatant of cell lysates and cell culture medium, while AEE was not detected. Therefore, the content change of AEE was expressed as the content change of its metabolite SA. In the uptake experiment, when the factors of time, concentration, and temperature were examined, the uptake of SA reached the maximum level within 30 min, and there was concentration dependence. In addition, low temperature (4°C) could significantly reduce the uptake of SA in Caco-2 cells. In the transport experiment, under the consideration of time, concentration, and temperature, the transepithelial transport of SA from AP-BL and BL-AP sides was time-dependent. The amount of SA transported in Caco-2 cells increased with the increase of concentration, but the transmembrane transport rate had no correlation with the concentration. This phenomenon may be due to the saturation phenomenon of high concentration. The efflux ratio (ER) was less than 1, which indicated that their intestinal transport mechanism was passive transport. Moreover, the temperature had a significant effect on the transport of AEE.

**Conclusion:** In summary, intestinal absorption of AEE through Caco-2 cell monolayers was related to passive transport. The uptake and transport of AEE were concentration-dependent, and temperature significantly affected their uptake and transport. The absorption and transport characteristics of AEE may contribute to the exploration of mechanisms of absorption and transport of chemosynthetic drugs *in vitro*.

## 1 Introduction

The oral administration of drugs is one of the most convenient routes of administration, and the small intestine is the most important site for oral drug absorption (Jørgensen et al., 2021). The apparent permeability coefficient (Papp) is an important parameter of bioavailability (Chi et al., 2019). Therefore, the absorption of the drug in the small intestine can be evaluated by important parameters such as the Papp of the substance in the *in vitro* model. In recent years, the Caco-2 cell monolayer model has been widely used to study the absorption of drugs *in vitro* (Gerasimenko et al., 2016) and is an effective *in vitro* model for investigating drug uptake and transport (Yee, 1997; Artursson et al., 2001; He et al., 2003; Fujikawa et al., 2005; Jin and Di, 2008).

Aspirin eugenol ester (AEE) is a novel medicinal compound synthesized by esterification of aspirin with eugenol using the prodrug principle. AEE has pharmacological activities against inflammation (Ma et al., 2017a), oxidative stress (Huang et al., 2019a; Huang et al., 2019b; Zhang et al., 2020a; Zhang et al., 2021), hypolipidemia (Karam et al., 2015; Karam et al., 2016), atherosclerosis (Ma et al., 2017b; Ma et al., 2017c), thrombosis (Ma et al., 2016; Shen et al., 2019), and acute liver injury (Zhang et al., 2020b). However, it has low water solubility and poor bioavailability. The previous studies showed that after oral administration in rats and dogs, the plasma concentrations of AEE and its prodrugs aspirin and eugenol were extremely low, while the concentration of the metabolite salicylic acid (SA) was relatively high (Shen et al., 2015). Many studies focused on the pharmacological and toxicological mechanisms of AEE, but the characteristics were still unclear on the absorption of AEE after oral administration. Therefore, the purpose of this study was to investigate the mechanisms of uptake and transport of AEE *in vitro* through Caco-2 cell monolayers and to examine the effects of time, concentration, and temperature on its uptake and transport. It provides the basis for the research and development of AEE preparation.

## 2 Materials and Methods

### 2.1 Chemicals and Materials

Caco-2 cells were obtained from American Type Culture Collection (ATCC, Manassas, VA, United States). AEE with a purity of 99.5% was prepared in the Lanzhou Institute of Husbandry and Pharmaceutical Sciences of CAAS, Lanzhou, China. Salicylic acid was purchased from the National Institutes for Food and Drug Control (Beijing, China). Trypsin, modified Eagle medium (MEM), Hanks buffer (HBSS), non-essential amino acids (NEAA), glutamine, sodium pyruvate 100 mM solution, and fetal bovine serum were obtained from Gibco (Grand Island, NY, United States). Cell counting kit-8 was purchased from Beyotime (Shanghai, China). BCA protein assay kit, phosphate-buffered saline (PBS), and lucifer yellow (LY) were supplied by Solarbio (Beijing, China). The alkaline phosphatase kit was purchased from Mlbio (Shanghai, China). Transwell-TM cell culture plate (0.4 μm, Model 3460) was purchased from Corning (United States). The other reagents of analytical grade were purchased from the Sinopharm Group (Shanghai, China).

### 2.2 Drug Pretreatment

AEE was dissolved in DMSO to form the stock solution of 256 mM. The stock solution was diluted with DMSO to get the solutions with different concentrations. The solutions were diluted by using MEM culture medium and HBSS to prepare the different concentrations for the cell viability assay and experiments of the uptake and transport, respectively.

### 2.3 Culture of Caco-2 Cells

Caco-2 cells were routinely maintained in 20% fetal bovine serum (FBS), 1% glutamine, 1% sodium pyruvate, 1% non-essential amino acids (NEAA), and 77% MEM media supplemented at 37°C under humidified atmospheric conditions containing 5% CO_2_ (Yang et al., 2013; Ye et al., 2013; Zheng et al., 2015). When reaching 80%∼90% confluency by observation under an inverted microscope, Caco-2 cells were trypsinized and passaged at a ratio of 1:3 (Kim et al., 2015); the Caco-2 cell suspension was seeded in T25 cell culture flasks with the mentioned medium.

### 2.4 Effect of Aspirin Eugenol Ester on the Activity of Caco-2 Cells

To test the cytotoxic effects of AEE on Caco-2 cells, the cell counting kit-8(CCK-8) assay (Beyotime, Shanghai, China) was performed (Zhang et al., 2021). Caco-2 cells were seeded in sterile 96-well culture plates at a density of 1 × 10^5^ cells per well (100 μl per well) and cultured for 24 h in medium (37°C, 5% CO_2_). The old medium was discarded. Then, 100 μl of AEE solution of different concentrations (16, 32, 64, 128, and 256 μM) was added, respectively. Six replicate wells were set up for each concentration. The cells were cultured for 24 h. A measure of 10 μl of CCK-8 solution was added to each well, and cells were cultured for 2 h (37°C, 5% CO_2_). Finally, the number of viable cells was assessed by measurement of the absorbance at 450 nm.

### 2.5 HPLC Analysis of Samples

The samples were processed for HPLC analysis. The samples were analyzed on an Agilent 1290 Infinity HPLC system (Agilent Technologies, United States) equipped with an ultraviolet detector. The chromatographic column was a C_18_ phase column (ODS, 150 mm × 4.6 mm, 5 μm, Dalian Elite Analytical Instruments) with gradient elution, mobile phase A (0.5% phosphoric acid), and mobile phase B (acetonitrile). From 0 to 10 min, mobile phase A was changed linearly from 60 to 40% and maintained until 30 min. From 30 to 35 min, mobile phase A was increased linearly from 40 to 60% and maintained until 40 min. The column temperature was 35°C. The detection wavelength was 279 nm. The flow rate was 1.0 ml min^−1^, and the injection volume was 10 µl.

Calibration curves were constructed within a range of 0.025–1 μM L^−1^. Samples of the resulting supernatants were collected and analyzed by HPLC. The peak areas of AEE and SA were measured, and the calibration curves of peak area vs. concentration and the coefficient were then analyzed.

### 2.6 Establishment of the Caco-2 Cell Monolayer Model

#### 2.6.1 Intake Experiment

Caco-2 cells (1×10^5^ cells/ml) were seeded in 12-well culture plates. Caco-2 cells were cultured under standard cell culture conditions and used in the uptake experiments after 2 weeks.

#### 2.6.2 Transport Experiment

Caco-2 cells (1×10^5^ cells/ml) were seeded in a 12-well Transwell-TM cell culture plate. A measure of 0.5 ml of cell suspension was transferred to the AP side. Then, 1.5 ml of MEM medium was transferred to the BL side. Caco-2 cells were cultured under standard cell culture conditions. The medium was replaced 24 h after initial inoculation. The cell culture medium was replaced every other day for the first week, and then, it was replaced every day. When cells were cultured for 22 consecutive days (Chen et al., 2016), their state was uniform and dense, forming a tightly connected monolayer of cells. It could be used for transport experiments.

### 2.7 Evaluation of the Caco-2 Cell Absorption Model

#### 2.7.1 Morphological Observation of Caco-2 Cells

Caco-2 cells were seeded in a 12-well Transwell-TM cell culture plate. The growth rate and morphology of the cells were observed using an inverted microscope daily, and pictures were taken at the appropriate time.

#### 2.7.2 Cell Transmembrane Resistance Detection

The appropriate number of days was chosen after the cells were seeded, and the TEER value of the Caco-2 cell monolayer model was measured using a cell resistance meter (MERS00002), and the changes were observed and recorded (Joseph et al., 2012; Xiang et al., 2020).

The value is calculated as follows:

$$\text{T}\text{E}\text{E}\text{R}\left(\Omega \cdot \text{c}{\text{m}}^{2}\right)=\left[\text{T}\text{E}\text{E}\text{R}\left(\Omega \right)-\text{T}\text{E}\text{E}{\text{R}}_{\text{b}\text{a}\text{c}\text{k}\text{g}\text{r}\text{o}\text{u}\text{n}\text{d}}\left(\Omega \right)\right]\times \text{A}\left(\text{c}{\text{m}}^{2}\right),$$

where A is the cell monolayer membrane area (1.12 cm^2^).

#### 2.7.3 Lucifer Yellow Transmission Rate Experiment

After treatments, the lucifer yellow transmission rate was assessed using the corresponding commercial kit, according to the manufacturer’s protocols. The Papp of the lucifer yellow in each plate was measured and calculated (Zha et al., 2009).

#### 2.7.4 Alkaline Phosphatase Activity Assay

The alkaline phosphatase kit was used to detect the enzyme activity on the AP and BL sides to determine whether the Caco-2 monolayer has differentiated (Wu et al., 2013).

### 2.8 Uptake and Transportation Experiments

#### 2.8.1 Transportation Experiment

Caco-2 cells were cultured for 22 days and formed a polarized monolayer before the start of the transportation experiment. The transportation experiment of AEE was divided into two phases, from the AP side to the BL side (AP-BL) and from the BL side to the AP side (BL-AP). To assess the transportation of AP-BL, 0.5 ml of different concentrations of the AEE solution was added to the AP side as the donor side, while 1.5 ml HBSS was added to the BL side as the recipient side. To assess the transportation of BL-AP, 1.5 ml of different concentrations of the AEE solution was added to the BL side as the donor side, while 0.5 ml HBSS was added to the AP side as the recipient side. Then, 200 μl of sample solution was collected on the recipient side in different time periods (15, 30, 45, 60, 90, and 120 min), and 200 μl of freshly preheated HBSS was added immediately. The concentrations of AEE and SA in samples were measured by HPLC. When studying the effect of temperature on the transport rate of AEE, the scheme was the same as mentioned earlier. The experiment was performed for 120 min.

The Papp is calculated according to the following formula, the unit is cm/s,

$${\text{P}}_{\text{a}\text{p}\text{p}}=\frac{\Delta Q/\Delta \text{t}}{\text{A}\cdot {\text{C}}_{0}}.$$

Δ*Q*/Δ*t* is the transport rate of AEE per unit time (μM/s), *A* is the micropore area of the polyester carbon film (1.12 cm^2^), and *C*_0_ is the initial concentration (μM) of AEE in the supply tank.

$$ER=\frac{P_{app}\left(BL-AP\right)}{P_{app}\left(AP-BL\right)}.$$

#### 2.8.2 Uptake Experiment

Caco-2 cells were cultured in 12-well plates for 14 days and used for drug uptake experiments. Cell metabolites were removed by three gentle washes with prewarmed HBSS 2 h before beginning the experiment. The cells were treated with different factors (the effects of time, concentration, and temperature). After treatments, 200 μl of the prechilled cell lysate was added to each well for 5 min to lyse the cells. The cells were scraped off by a cell scraper, and the lysate containing the broken cells was pipetted into an EP tube and centrifuged at 14,000 g for 5 min. The portion of the supernatant was collected and analyzed by HPLC. The bicinchoninic acid assay (BCA) was used to determine the total cell protein in the supernatant. The intake of AEE by Caco-2 cells was expressed as the amount of SA per milligram of protein.

### 2.9 Statistics and Analysis

Statistical analysis was carried out using the SAS 9.2 (SAS Institute Inc., NC, United States). All data are presented as means ± SD. The differences among different treatment groups were analyzed with one-way ANOVA, followed by Duncan’s multiple comparisons. Statistical significance was considered at *p* < 0.05.

## 3 Results

### 3.1 Cytotoxicity of Aspirin Eugenol Ester

**FIGURE 1:**
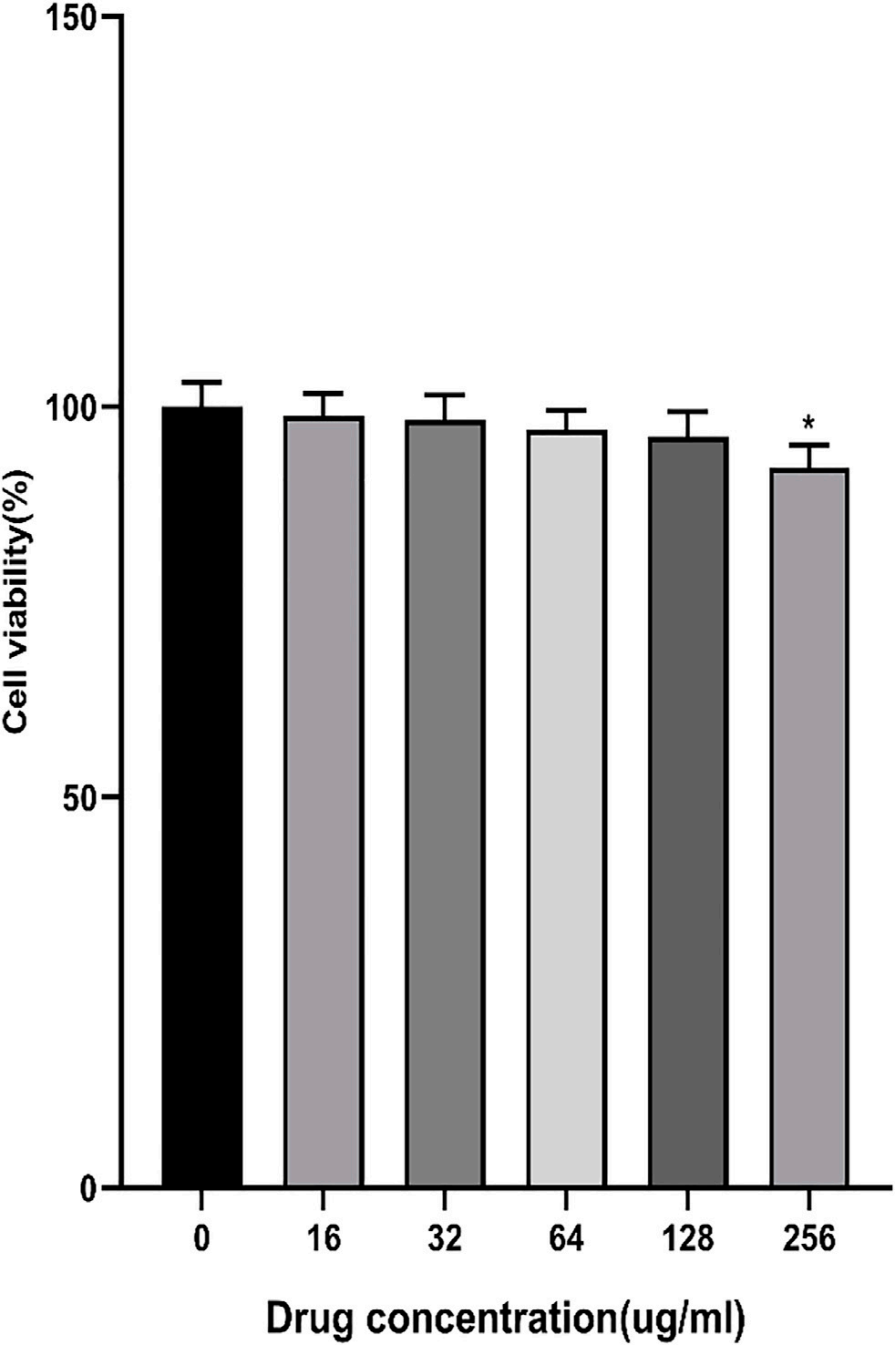
Effects of different concentrations of AEE (16∼256 μM) on Caco-2 cell viability. Values are presented as the means ± SD where applicable (*n* = 6). *p < 0.05 than the control group.

The results showed that AEE at16∼128 μM had no cytotoxicity to Caco-2 cells for 24 h. When the concentration was 256 μM, the cell viability decreased slightly (Figure 1). Therefore, the concentrations of AEE below 256 μM were selected for subsequent experiments.

### 3.2 Evaluation of the Caco-2 Cell Monolayer Model

#### 3.2.1 Observation of Caco-2 Cell Morphology

**FIGURE 2:**
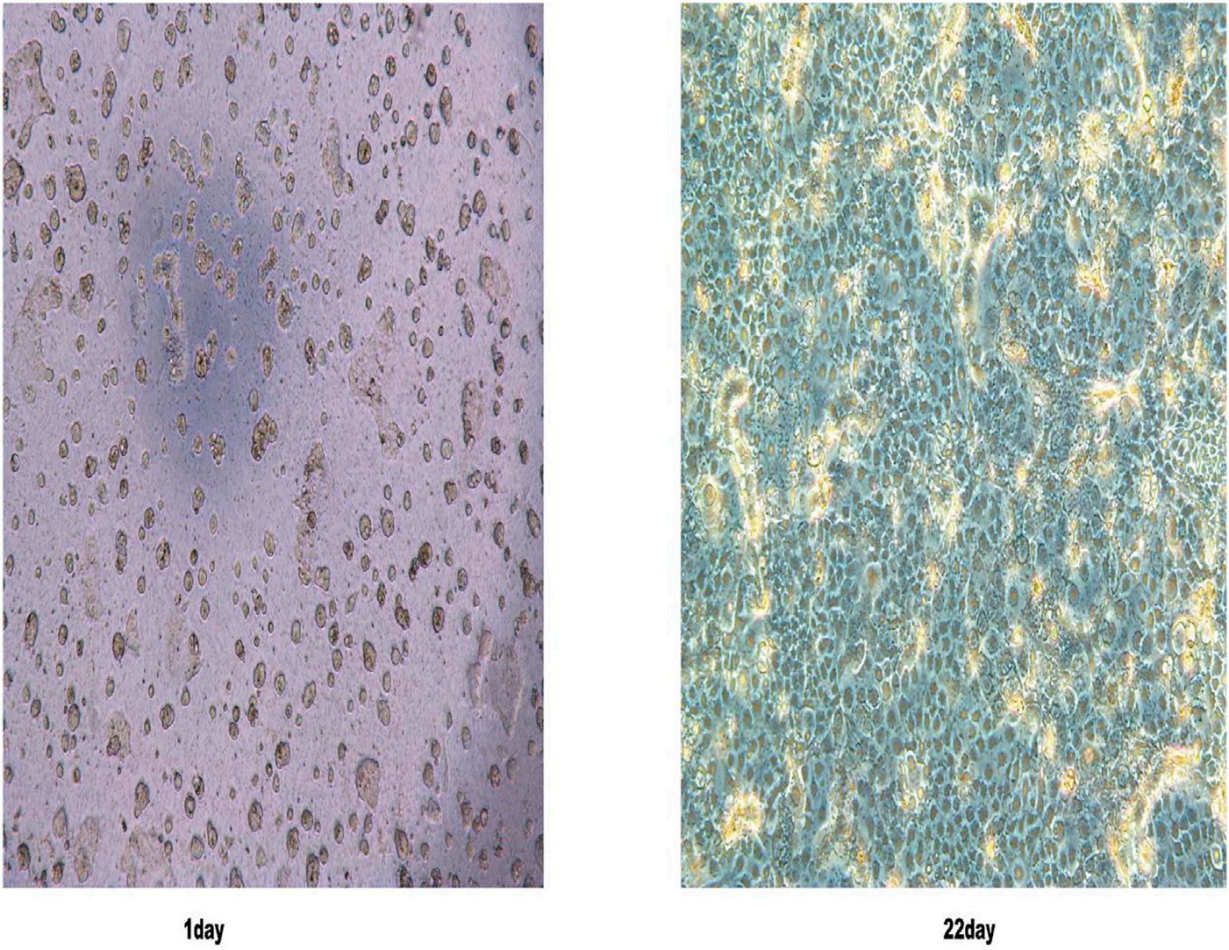
Caco-2 cell morphology was observed under an inverted light microscope on the first and 22nd day.

The growth morphology of Caco-2 cells was observed by using an optical inverted microscope. The results showed that when the cells were cultured continuously for 22 days, their state was uniform and dense, forming a single cell layer (Figure 2), indicating that the cell monolayer model had been successfully established.

#### 3.2.2 Transmembrane Resistance Detection

**FIGURE 3:**
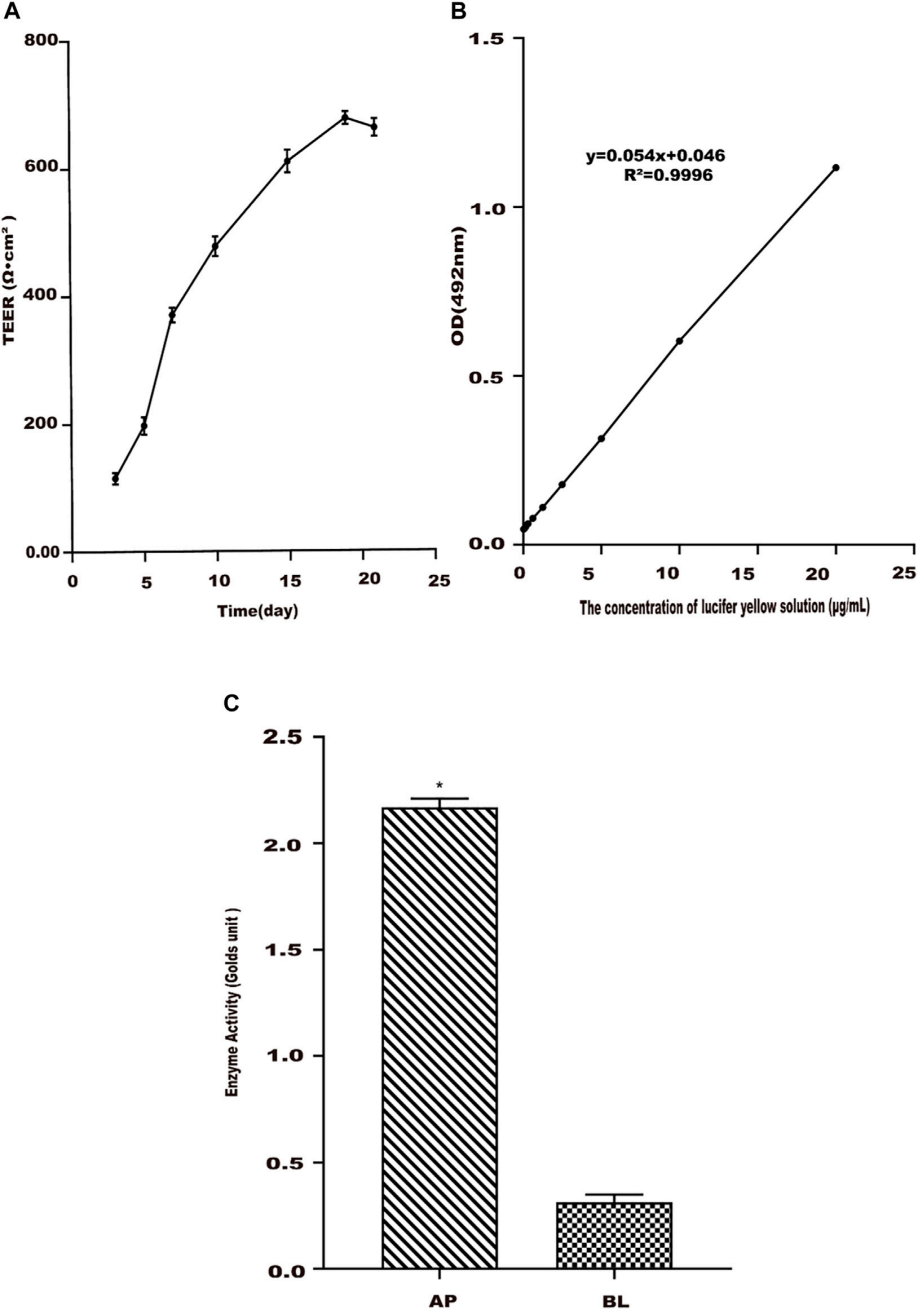
Establishment of the Caco-2 cell transport model. **(A)** Change trend of Caco-2 cell monolayer transmembrane resistance (TEER) with culture time. **(B)** Standard curve of lucifer yellow from the AP side to BL side in Caco-2 cells. **(C)** Alkaline phosphatase activities in apical (AP) and basolateral (BL) sides of Caco-2 cell monolayers. Values are presented as the means ± SD where applicable (n = 6). *p < 0.05 than the BL side.

Studies showed that there was a proportional relationship between the TEER value and the tight junctions of the Caco-2 cell monolayer (Duizer et al., 1999). From Figure 3A, TEER reached the best at 21 days, and the resistance values were greater than 500 Ω cm^2^, which showed that the monolayer membrane of Caco-2 cells has good compactness. Since TEER values entered a steady state (Xiang et al., 2020), the experiment was started on day 22.

#### 3.2.3 Lucifer Yellow Transmission Rate

Due to the poor transport and absorption of LY solution in the Caco-2 cell monolayer model, it is often used as a negative control drug to test the integrity of the Caco-2 cell monolayer model. The linear relationship of LY between the concentration of 0.3–20 μg/ml and the absorbance value was good (Figure 3B). Its regression equation was *y* = 0.0539*x* + 0.0464 (*R* ^2^ = 0.9996). A measure of 20 μg/ml of the LY solution was added to the cell compartment, and the absorbance was measured after 2 h. The Papp of LY with cells was determined as 0.37 × 10^–6^ cm s^−1^ in the study, less than 0.5 × 10^–6^ cm s^−1^ (Artursson et al., 2001). This result showed that the compactness of the Caco-2 cell monolayer was good, meeting the experimental requirements.

#### 3.2.4 Alkaline Phosphatase Activity

The activity of alkaline phosphatase was tested when Caco-2 cells were cultured for 22 days. The results were shown in Figure 3C. The alkaline phosphatase activity on the AP side of the cell monolayer was higher than that on the BL side, and its activity value was about 12 times that of the latter. It meant that the alkaline phosphatase distribution was extremely asymmetric (Billat et al., 2017; Birch et al., 2018), indicating that the cells have been polarized.

### 3.3 Uptake Experiment

#### 3.3.1 Effect of Time on the Uptake of Aspirin Eugenol Ester

**FIGURE 4:**
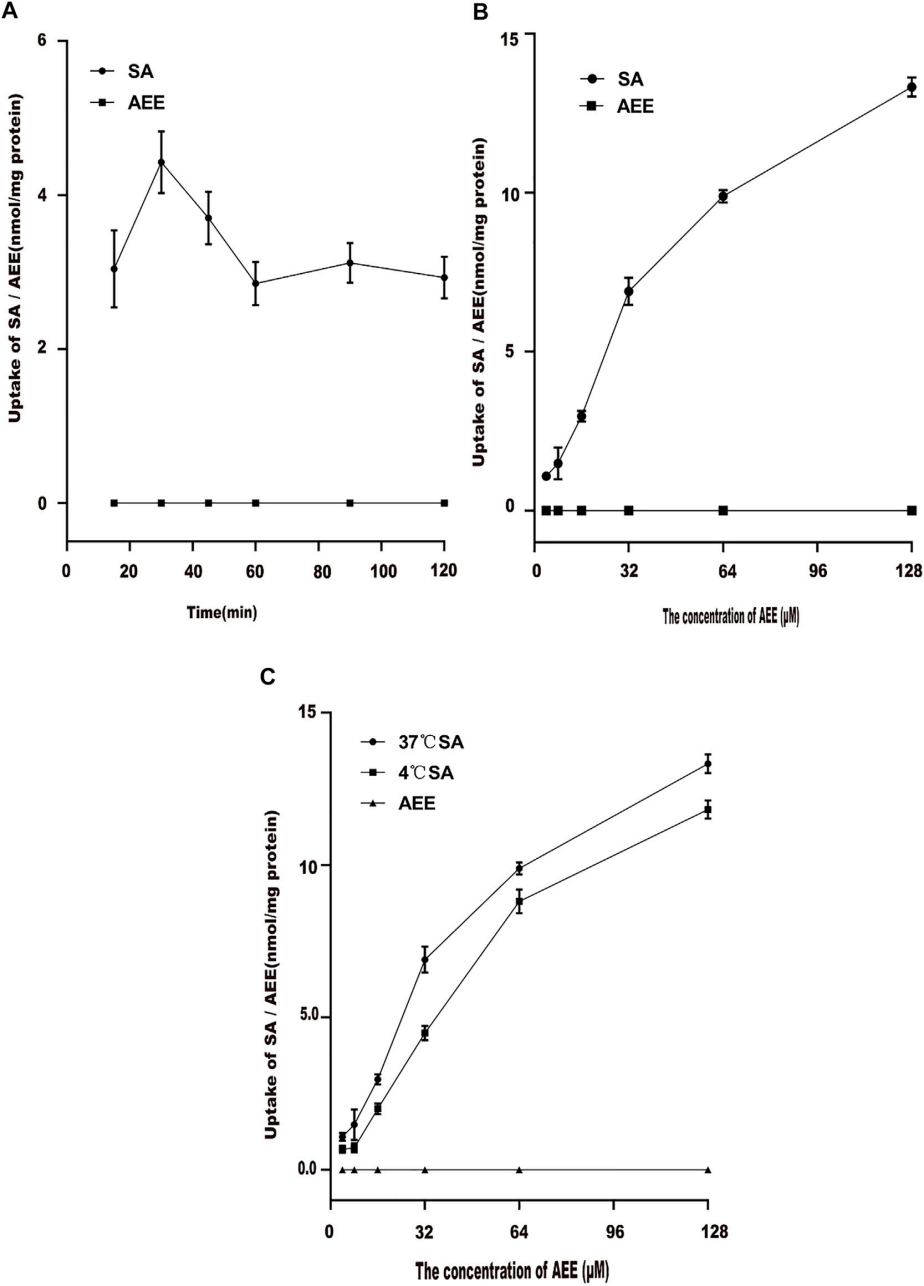
Effect of different factors on the uptake of AEE in the Caco-2 cell model. The main active metabolite of AEE as salicylic acid represented for AEE was quantified after administration. **(A)** Effect of times on the uptake of AEE (64 μM). **(B)** Effect of concentrations on the uptake of AEE during 30 min. **(C)** Effect of temperature (37°C and 4°C) on the uptake of AEE during 30 min. Values are presented as the means ± SD where applicable (*n* = 6). *p < 0.05 compared with 4°C.

The effect of time on AEE uptake in Caco-2 cells was studied. The results are shown in Figure 4A. Higher concentrations of SA were detected in the supernatant of the cell lysate, but AEE was not detected. Therefore, the change in the content of its metabolite SA represented the amount of intracellular AEE. The uptake of SA in Caco-2 cells reached the maximum level at 30 min. When the time exceeded 30 min, the intake suddenly dropped. Therefore, the time for the subsequent uptake experiment was chosen to be 30 min.

#### 3.3.2 Effect of Concentration on the Uptake of Aspirin Eugenol Ester

Different concentrations of AEE were added to Caco-2 cells for 30 min to explore the effect of drug concentration on cell uptake. The results are shown in Figure 4B. When the concentration range was 4∼32 μM, the uptake of Caco-2 cells increased rapidly. The uptake of Caco-2 cells increased slowly at 64∼128 μM. This may be due to the saturation of the uptake of Caco-2 cells.

#### 3.3.3 Effect of Temperature on the Uptake of Aspirin Eugenol Ester

The effect of temperature on AEE uptake in Caco-2 cells was studied. After Caco-2 cells were incubated with 64 μM AEE for 30 min, the effects of different temperatures on the uptake of AEE in Caco-2 cells were determined. The results are shown in Figure 4C. When the temperature changed from 37°C to 4°C, the uptake of Caco-2 cells changed significantly. The intake at 37°C was higher than that at 4°C. The results showed that as the temperature decreased, the uptake of SA decreased significantly, which may be due to the lower temperature affecting the fluidity of the cell membrane, thereby reducing the uptake of SA.

### 3.4 Transport Experiment

#### 3.4.1 Effect of Time and Temperature on the Transport of Aspirin Eugenol Ester

**FIGURE 5:**
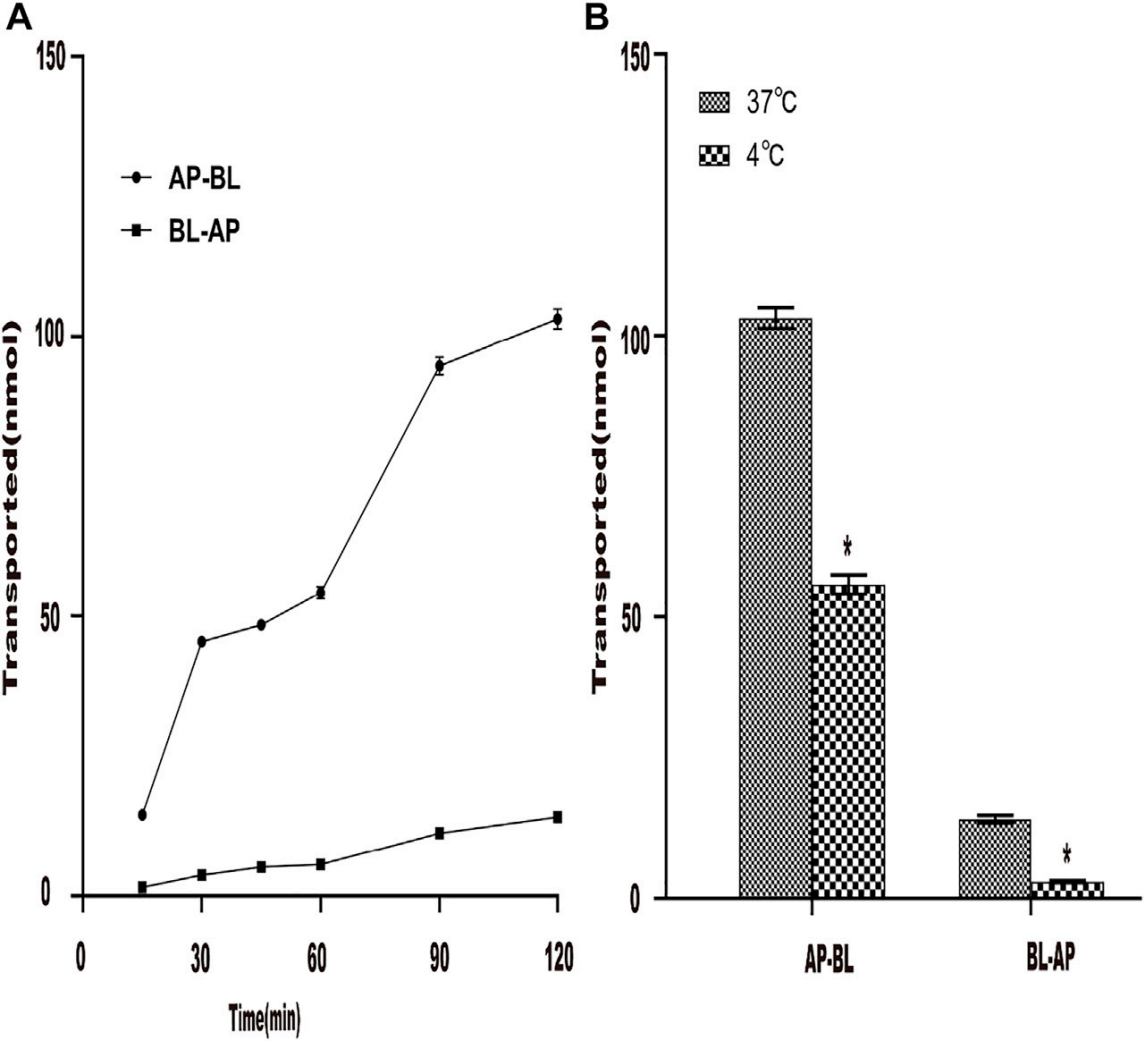
Effect of time and temperature on the transportation of AEE in the Caco-2 cell model. The main active metabolite of AEE as salicylic acid represented for AEE was quantified after administration. **(A)** Effect of time (120 min) on the transport of AEE on both sides of AP-BL and BL-AP in Caco-2 cells at 37°C. **(B)** Effect of temperature (37°C and 4°C) on the transport of AEE on both sides of AP-BL and BL-AP in Caco-2 cells during 120 min. Values are presented as the means ± SD where applicable (*n* = 6). *p < 0.05 than the BL side.

First, the effect of time on the transport of AEE in Caco-2 cells was studied. The results are shown in Figure 5A. At 37°C, the AEE concentration of the Caco-2 cell monolayer was constant. Within 120 min, the transport volume of SA increased with time but did not reach saturation. The results showed that the transepithelial transport of SA from the AP-BL and BL-AP sides was time-dependent, indicating that SA could be transported through epithelial cells. In addition, when the temperature was lowered from 37°C to 4°C, the transport of SA in Caco-2 cells was significantly reduced, indicating that the transport of SA was related to temperature (Figure 5B).

#### 3.4.2 Total Transmembrane Transport of Aspirin Eugenol Ester on Both Sides of Caco-2 Cells

**FIGURE 6:**
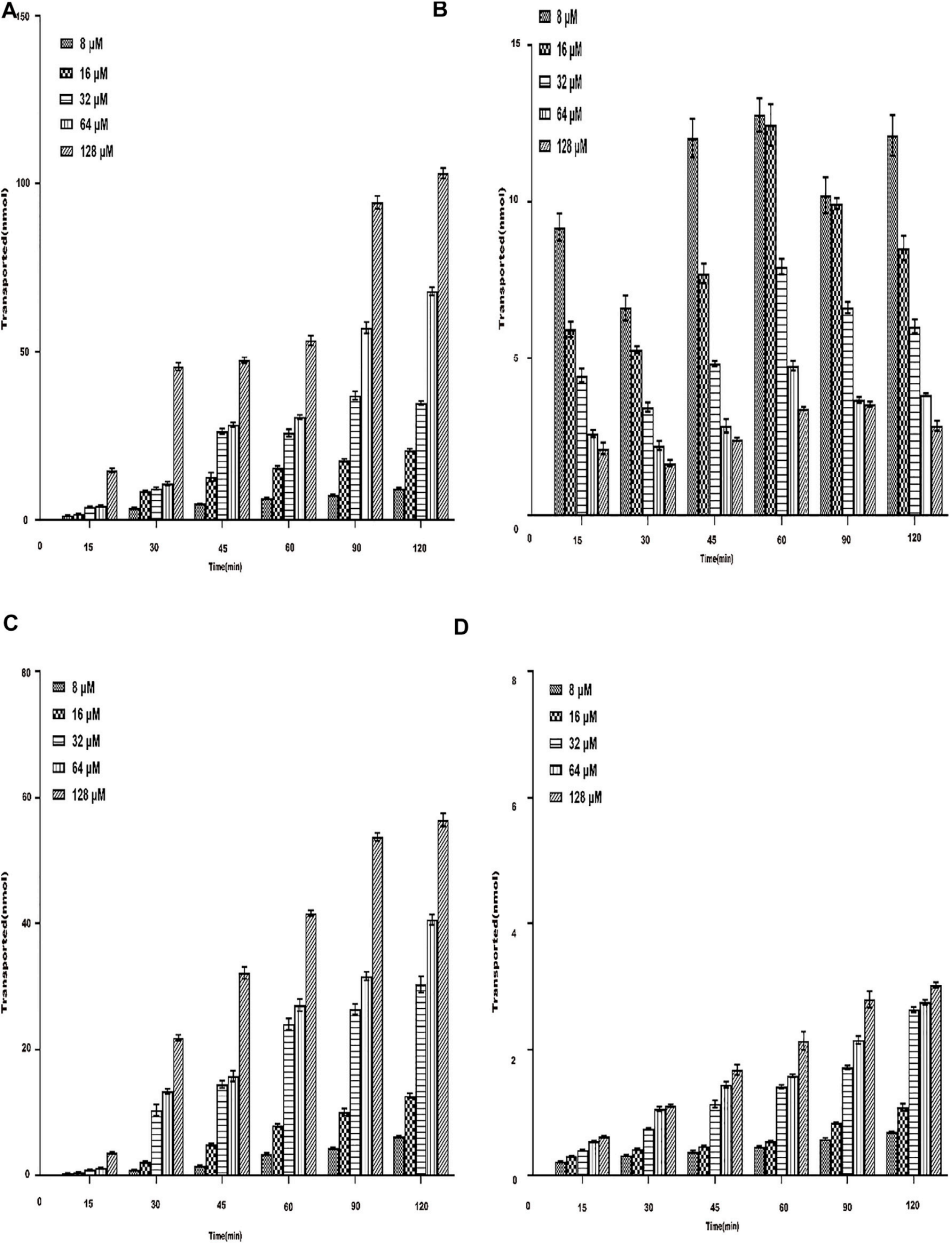
Total transmembrane transport of AEE with different concentrations (8∼128 μM) on both sides of Caco-2 cells. The main active metabolite of AEE as salicylic acid represented for AEE was quantified after administration. **(A)** AP-BL at 37°C. **(B)** BL-AP at 37°C. **(C)** AP-BL at 4°C. **(D)** BL-AP at 4°C.Values are presented as the means ± SD where applicable (*n* = 6). *p < 0.05 than the BL side.

Under the condition of 37°C, the transport amount of AEE in Caco-2 cells increased with the increase in the concentration, and the transmembrane transport rate was related to the concentration. The results are shown in Figures 6A,B. The results showed that when AEE of different concentrations were transported across the AP-BL side and BL-AP side, the amount of SA transported gradually increased within 15∼120 min. The membrane transport rate was also time-dependent. Interestingly, when the transport experiment time was between 90 and 120 min, the increase in transport volume on the AP-BL side and the BL-AP side was slower. It did not reach full saturation yet. The results showed that the transport capacity of SA on the AP side was higher than that on the BL side. This transport experiment was performed again at 4°C. The results are shown in Figures 6C,D. The results showed that both the AP-BL side and the BL-AP side of the transport volume were less than 37°C, which indicated that temperature had a significant effect on the transport of SA in Caco-2 cells.

#### 3.4.3 Apparent Permeability Coefficient (Papp) of Transmembrane Transport of Aspirin Eugenol Ester

**FIGURE 7:**
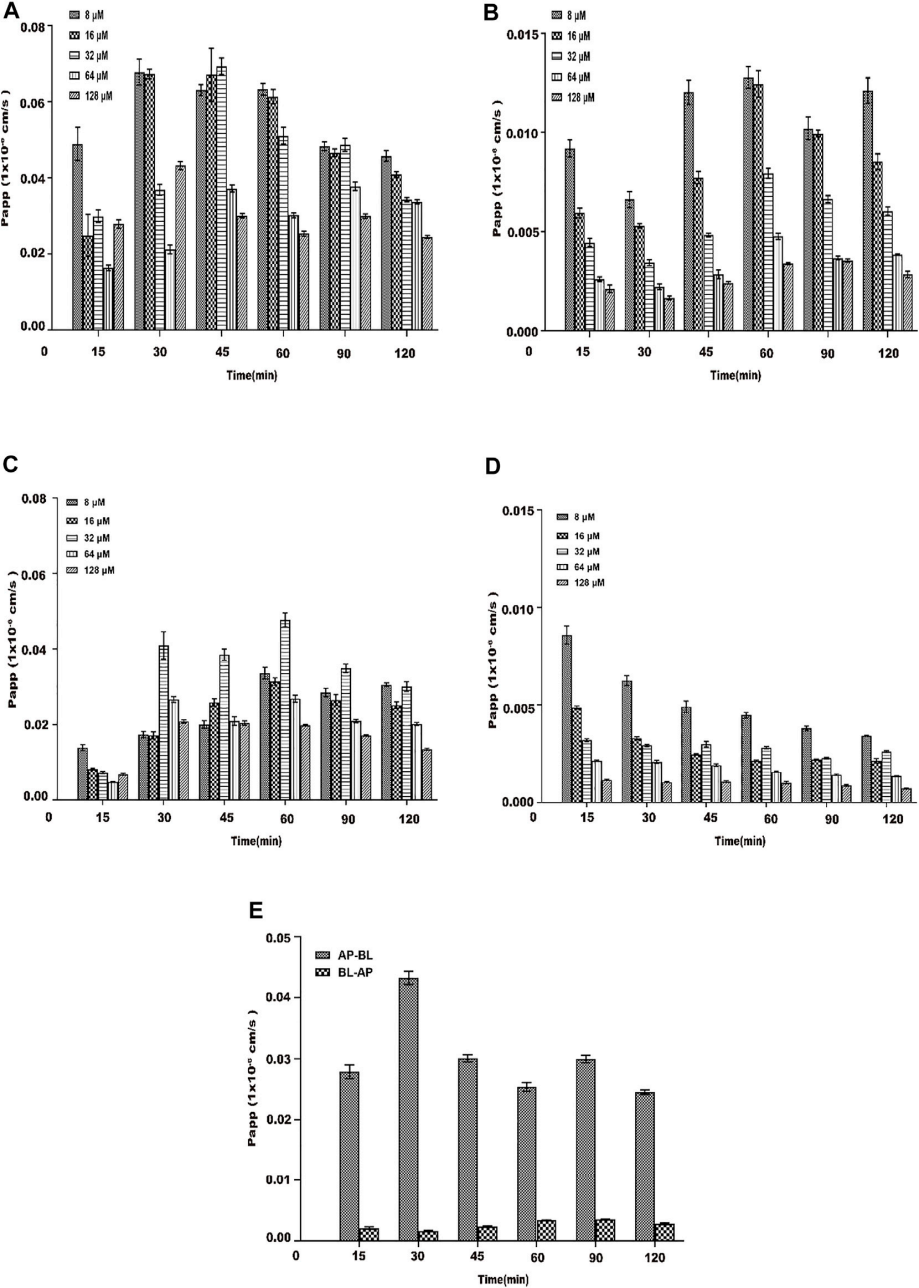
Papp of each concentration (8∼128 μM) of AEE during 120 min. The main active metabolite of AEE as salicylic acid represented for AEE was quantified after administration. **(A)** From the AP side to BL side at 37°C. **(B)** From the BL side to AP side at 37°C. **(C)** From the AP side to BL side at 4°C. **(D)** From the BL side to AP side at 4°C.**(E)** AP-BL than BL-AP (64 μM) at 37°C. Values are presented as the means ± SD where applicable (*n* = 6). *p < 0.05 than the BL side.

The Papp of the drug through the Caco-2 cell monolayer model can better reflect its absorption *in vivo* (Turco et al., 2011; Fu et al., 2015), and the Papp value can also reflect the ability of the substance to be absorbed in the intestine (Artursson et al., 2001). The Papp was examined from the AP side to BL side and BL side to AP side within 120 min for different concentrations of AEE (8∼128 μM) under the condition of 37°C. From Figure 7A, during the transportation of different concentrations of AEE on the AP-BL side, the Papp of each concentration group and each time group increased slowly within the range of 15∼30 min. The Papp value of the low-concentration group was higher than that of the high-concentration group, which may be due to saturation in the high-concentration group. In the range of 45–120 min, the Papp value of each concentration group and each time group decreased sequentially. In the process of BL-AP side transport, the Papp value of each concentration group gradually increased with the increase in time (15∼60 min), and the Papp value of each concentration group in the range of 60∼120 min gradually decreased with the increase in time. The Papp value of each time group gradually decreased with the increase in the concentration (8∼128 μM) (seen in Figure 7B). Similarly, the investigations were performed at 4°C. From Figures 7C,D, temperature had a significant effect on the transport and absorption of SA. From Figure 7E, the Papp of AEE (64 μM) from AP to BL was determined to be 0.034 (±0.012)×10^–6^ cm⋅s^−1^, which was considered to have poor permeability and absorptive *in vivo* rate.

## 4 Discussion

AEE is a new type of potential medicinal compound with activities such as anti-inflammatory, anti-oxidant, alleviation of hyperlipidemia, prevention of atherosclerosis, prevention of thrombosis, and anti-acute liver injury. However, due to poor water solubility, the *in vivo* bioavailability is low, and its intestinal absorption and transport characteristics are still unknown.

The Caco-2 cell monolayer model has been established successfully for investigating intestinal absorption and transport characteristics of drugs. The Caco-2 cell monolayer model is evaluated through the following four indicators: cell morphology, cell transmembrane resistance, alkaline phosphatase, and LY transmittance. The results showed that Caco-2 cells were tightly connected when cultured for 22 days, TEER > 500 Ω cm^2^, LY permeability <1%, and alkaline phosphatase activity distribution was extremely asymmetrical. These results indicated that the Caco-2 cell model was suitable for studying the mechanism of AEE intestinal uptake and transport (Xiang et al., 2018; Dou et al., 2019; Li et al., 2021).

The purpose of this study was to explore the uptake and transport mechanism of AEE in the Caco-2 cell monolayer model. The results showed that several factors (time, concentration, and temperature) affected its absorption and transport. A higher concentration of SA was detected in the supernatant of the cell lysate and cell culture fluid, but AEE was not detected, indicating that AEE was metabolized to produce metabolite SA during the uptake and transport process. This was consistent with the results of previous studies (Shen et al., 2015). Therefore, the change in the content of its metabolite SA represented the change in the content of AEE. As shown in the time and concentration results, the uptake and transport of AEE were time- and concentration-dependent. The effect of time and concentration on the uptake and transport of FITC-PoIFN-α in Caco-2 cells was studied, and the results showed that the uptake and transport of FITC-PoIFN-α were time- and concentration-dependent (Liu et al., 2017). The present results were consistent with them, suggesting that uptake and transport of AEE were related to time and concentration. In the uptake experiment, the absorption of SA in Caco-2 cells reached the maximum level at 30 min. The effect of time on the uptake of dihydromyricetin in Caco-2 cells was studied, and the results showed that the uptake of dihydromyricetin was related to time, increased first, and then reached saturation (Xiang et al., 2018). Our results were consistent with them, suggesting that uptake of AEE was related to time. In addition, temperature also had a significant effect on it. The efflux ratio is the quotient of the apparent permeability in the BL-AP direction and the AP-BL direction (Anand et al., 2008; Sun et al., 2008). In the transport experiment, the efflux ratio was much lower than 1.0. The result indicated that the secretion rate of AEE was much slower than the absorption rate. The absorption carrier on the AP side might participate in the transport of AEE, which illustrated that AEE consumed energy in the transport process of Caco-2 cells (Qiang et al., 2011). These results suggested that in addition to passive transport, AEE might also undergo active transport. The drugs are transported from the AP side to the BL side into the systemic circulation and then exert their efficacy. Therefore, the determination of the Papp is necessary to predict the permeability and oral absorption of the drugs (Ma et al., 2014; Abuhelwa et al., 2016). The results suggested that the Papp of AEE (64 μM) from the AP to BL direction was determined to be 0.034 (±0.012)×10^–6^ cm s^−1^. According to the current international standards for material absorption (Wahlström and Blennow, 1978; Walters et al., 2000), the bioavailability is 70%∼100%, and the Papp value is greater than 10 × 10^–6^ cm s^−1^, indicating good absorption. The bioavailability is 20%∼70%, and the Papp value is 1.0 × 10^–6^∼1.0 × 10^–5^ cm s^−1^, which is moderate absorption. The bioavailability is 0%∼20%, and the Papp value < 1.0 × 10^–6^ cm s^−1^, indicating a poorly absorbed substance. Therefore, AEE was considered to have poor permeability and absorptive *in vivo* rate (Hubatsch et al., 2007). Similarly, the same experiment was performed at 4°C. The results further illustrated that temperature had a significant effect on the transport and absorption of SA. The effect of temperature on the transport of aconitine in Caco-2 cells was studied, and the experiments also were set at 37°C and 4°C, respectively. The results showed that the transport of aconitine was related to temperature (Yang et al., 2013). Our results were consistent with that, indicating that the transport of AEE was related to temperature. In the subsequent research, the molecular mechanism of AEE such as the relationship between cellular transport efficiency and channel proteins will be explored, which can make the obtained results more valuable.

## 5 Conclusion

The monolayer culture model of Caco-2 cells was successfully constructed and applied to the study of AEE absorption *in vitro*. The effects of time, concentration, and temperature on the transport and uptake of AEE across the Caco-2 cell monolayer were studied to clarify the intestinal absorption mechanism of AEE. The results indicated that in addition to the passive transfer of AEE during the transfer process, there may be active transfer. The uptake and transport of AEE were time- and concentration-dependent, and temperature had a significant effect on it. These results confirmed the absorption characteristics of AEE and provided a reasonable explanation for the poor absorption of AEE observed in pharmacokinetics studies.

## Data Availability

The original contributions presented in the study are included in the article/Supplementary Material, further inquiries can be directed to the corresponding authors.

## Abbreviations

AEE, aspirin eugenol ester; AP side, side of the intestine; BCA, bicinchoninic acid; BL side, lower chamber is the basal side; CCK-8, cell counting kit-8; DMSO, dimethyl sulfoxide; ER, efflux ratio; FBS, fetal bovine serum; HBSS, Hanks buffer; LY, Lucifer yellow; MEM, modified eagle medium; NEAA, non-essential amino acids; Papp, apparent permeability coefficient; SA, salicylic acid; TEER, transepithelial electrical resistance.

## References

[B1] Abuhelwa A. Y. Foster D. J. R. Upton R. N. (2016). A Quantitative Review and Meta-Models of the Variability and Factors Affecting Oral Drug Absorption-Part I: Gastrointestinal pH. AAPS J. 18 (5), 1309–1321. 10.1208/s12248-016-9952-8 27495120

[B2] Anand P. Sundaram C. Jhurani S. Kunnumakkara A. B. Aggarwal B. B. (2008). Curcumin and Cancer: an "Old-Age" Disease with an "Age-Old" Solution. Cancer Lett. 267 (1), 133–164. 10.1016/j.canlet.2008.03.025 18462866

[B3] Artursson P. Palm K. Luthman K. (2001). Caco-2 Monolayers in Experimental and Theoretical Predictions of Drug Transport. Adv. Drug Deliv. Rev. 46 (1-3), 27–43. 10.1016/s0169-409x(00)00128-9 11259831

[B4] Billat P. A. Roger E. Faure S. Lagarce F. (2017). Models for Drug Absorption from the Small Intestine: where Are We and where Are We Going? Drug Discov. Today 22 (5), 761–775. 10.1016/j.drudis.2017.01.007 28115264

[B5] Birch D. Diedrichsen R. G. Christophersen P. C. Mu H. Nielsen H. M. (2018). Evaluation of Drug Permeation under Fed State Conditions Using Mucus-Covered Caco-2 Cell Epithelium. Eur. J. Pharm. Sci. 118, 144–153. 10.1016/j.ejps.2018.02.032 29524592

[B6] Chen L. Lu X. Liang X. Hong D. Guan Z. Guan Y. (2016). Mechanistic Studies of the Transport of Peimine in the Caco-2 Cell Model. Acta Pharm. Sin B 6 (2), 125–131. 10.1016/j.apsb.2016.01.006 27006896 PMC4788709

[B7] Chi C. T. Lee M. H. Weng C. F. Leong M. K. (2019). In Silico Prediction of PAMPA Effective Permeability Using a Two-QSAR Approach. Int. J. Mol. Sci. 20 (13), 3170. 10.3390/ijms20133170 PMC6651837 31261723

[B8] Dou T. Wang J. Han C. Shao X. Zhang J. Lu W. (2019). Cellular Uptake and Transport Characteristics of Chitosan Modified Nanoparticles in Caco-2 Cell Monolayers. Int. J. Biol. Macromol 138, 791–799. 10.1016/j.ijbiomac.2019.07.168 31356947

[B9] Duizer E. Gilde A. J. Versantvoort C. H. Groten J. P. (1999). Effects of Cadmium Chloride on the Paracellular Barrier Function of Intestinal Epithelial Cell Lines. Toxicol. Appl. Pharmacol. 155 (2), 117–126. 10.1006/taap.1998.8589 10053166

[B10] Fu Q. Wang H. Xia M. Deng B. Shen H. Ji G. (2015). The Effect of Phytic Acid on Tight Junctions in the Human Intestinal Caco-2 Cell Line and its Mechanism. Eur. J. Pharm. Sci. 80, 1–8. 10.1016/j.ejps.2015.09.009 26385515

[B11] Fujikawa M. Ano R. Nakao K. Shimizu R. Akamatsu M. (2005). Relationships between Structure and High-Throughput Screening Permeability of Diverse Drugs with Artificial Membranes: Application to Prediction of Caco-2 Cell Permeability. Bioorg. Med. Chem. 13 (15), 4721–4732. 10.1016/j.bmc.2005.04.076 15936203

[B12] Gerasimenko T. N. Senyavina N. V. Anisimov N. U. Tonevitskaya S. A. (2016). A Model of Cadmium Uptake and Transport in Caco-2 Cells. Bull. Exp. Biol. Med. 161 (1), 187–192. 10.1007/s10517-016-3373-7 27259497

[B13] He X. Sugawara M. Kobayashi M. Takekuma Y. Miyazaki K. (2003). An *In Vitro* System for Prediction of Oral Absorption of Relatively Water-Soluble Drugs and Ester Prodrugs. Int. J. Pharm. 263 (1-2), 35–44. 10.1016/s0378-5173(03)00343-0 12954178

[B14] Huang M. Z. Yang Y. J. Liu X. W. Qin Z. Li J. Y. (2019). Aspirin Eugenol Ester Attenuates Oxidative Injury of Vascular Endothelial Cells by Regulating NOS and Nrf2 Signalling Pathways. Br. J. Pharmacol. 176 (7), 906–918. 10.1111/bph.14592 30706438 PMC6433644

[B15] Huang M. Z. Yang Y. J. Liu X. W. Qin Z. Li J. Y. (2019). Aspirin Eugenol Ester Reduces H2O2-Induced Oxidative Stress of HUVECs via Mitochondria-Lysosome Axis. Oxid Med. Cel Longev 2019, 8098135. 10.1155/2019/8098135 PMC6754946 31583045

[B16] Hubatsch I. Ragnarsson E. G. Artursson P. (2007). Determination of Drug Permeability and Prediction of Drug Absorption in Caco-2 Monolayers. Nat. Protoc. 2 (9), 2111–2119. 10.1038/nprot.2007.303 17853866

[B17] Jin H. Di L. (2008). Permeability--In Vitro Assays for Assessing Drug Transporter Activity. Curr. Drug Metab. 9 (9), 911–920. 10.2174/138920008786485056 18991588

[B18] Jørgensen J. R. Thamdrup L. H. E. Kamguyan K. Nielsen L. H. Nielsen H. M. Boisen A. (2021). Design of a Self-Unfolding Delivery Concept for Oral Administration of Macromolecules. J. Control. Release 329, 948–954. 10.1016/j.jconrel.2020.10.024 33086101

[B19] Joseph M. M. Aravind S. R. Varghese S. Mini S. Sreelekha T. T. (2012). Evaluation of Antioxidant, Antitumor and Immunomodulatory Properties of Polysaccharide Isolated from Fruit Rind of Punica Granatum. Mol. Med. Rep. 5 (2), 489–496. 10.3892/mmr.2011.638 22012001

[B20] Karam I. Ma N. Liu X. W. Kong X. J. Zhao X. L. Yang Y. J. (2016). Lowering Effects of Aspirin Eugenol Ester on Blood Lipids in Rats with High Fat Diet. Lipids Health Dis. 15 (1), 196. 10.1186/s12944-016-0369-2 27855711 PMC5114728

[B21] Karam I. Ma N. Liu X. W. Li S. H. Kong X. J. Li J. Y. (2015). Regulation Effect of Aspirin Eugenol Ester on Blood Lipids in Wistar Rats with Hyperlipidemia. BMC Vet. Res. 11, 217. 10.1186/s12917-015-0523-5 26289078 PMC4546030

[B22] Kim S. Shin B. S. Ma E. (2015). Synthesis and Caco-2 Cell Permeability of N-Substituted Anthranilamide Esters as ADP Inhibitor in Platelets. Arch. Pharm. Res. 38 (6), 1147–1156. 10.1007/s12272-014-0353-1 25325926

[B23] Li F. Wei Y. Zhao J. Yu G. Huang L. Li Q. (2021). Transport Mechanism and Subcellular Localization of a Polysaccharide from Cucurbia Moschata across Caco-2 Cells Model. Int. J. Biol. Macromol 182, 1003–1014. 10.1016/j.ijbiomac.2021.04.107 33892025

[B24] Liu X. Zheng S. Qin Y. Ding W. Tu Y. Chen X. (2017). Experimental Evaluation of the Transport Mechanisms of PoIFN-α in Caco-2 Cells. Front. Pharmacol. 8, 781. 10.3389/fphar.2017.00781 29163167 PMC5681924

[B25] Ma N. Liu X. Kong X. Li S. Jiao Z. Qin Z. (2017). Feces and Liver Tissue Metabonomics Studies on the Regulatory Effect of Aspirin Eugenol Eater in Hyperlipidemic Rats. Lipids Health Dis. 16 (1), 240. 10.1186/s12944-017-0633-0 29228968 PMC5725792

[B26] Ma N. Liu X. W. Yang Y. J. Shen D. S. Zhao X. L. Mohamed I. (2016). Evaluation on Antithrombotic Effect of Aspirin Eugenol Ester from the View of Platelet Aggregation, Hemorheology, TXB2/6-Keto-Pgf1α and Blood Biochemistry in Rat Model. BMC Vet. Res. 12 (1), 108. 10.1186/s12917-016-0738-0 27296110 PMC4907079

[B27] Ma N. Yang G. Z. Liu X. W. Yang Y. J. Mohamed I. Liu G. R. (2017). Impact of Aspirin Eugenol Ester on Cyclooxygenase-1, Cyclooxygenase-2, C-Reactive Protein, Prothrombin and Arachidonate 5-Lipoxygenase in Healthy Rats. Iran J. Pharm. Res. 16 (4), 1443–1451. 29552053 PMC5843306

[B28] Ma N. Yang Y. Liu X. Kong X. Li S. Qin Z. (2017). UPLC-Q-TOF/MS-based Metabonomic Studies on the Intervention Effects of Aspirin Eugenol Ester in Atherosclerosis Hamsters. Sci. Rep. 7 (1), 10544. 10.1038/s41598-017-11422-7 28874840 PMC5585262

[B29] Ma Y. Zeng M. Sun R. Hu M. (2014). Disposition of Flavonoids Impacts Their Efficacy and Safety. Curr. Drug Metab. 15 (9), 841–864. 10.2174/1389200216666150206123719 25658129

[B30] Qiang Z. Ye Z. Hauck C. Murphy P. A. McCoy J. A. Widrlechner M. P. (2011). Permeability of Rosmarinic Acid in Prunella Vulgaris and Ursolic Acid in Salvia Officinalis Extracts across Caco-2 Cell Monolayers. J. Ethnopharmacol 137 (3), 1107–1112. 10.1016/j.jep.2011.07.037 21798330 PMC3202029

[B31] Shen D. S. Yang Y. J. Kong X. J. Ma N. Liu X. W. Li S. H. (2019). Aspirin Eugenol Ester Inhibits Agonist-Induced Platelet Aggregation *In Vitro* by Regulating PI3K/Akt, MAPK and Sirt 1/CD40L Pathways. Eur. J. Pharmacol. 852, 1–13. 10.1016/j.ejphar.2019.02.032 30797789

[B32] Shen Y. Liu X. Yang Y. Li J. Ma N. Li B. (2015). *In Vivo* and *In Vitro* Metabolism of Aspirin Eugenol Ester in Dog by Liquid Chromatography Tandem Mass Spectrometry. Biomed. Chromatogr. 29 (1), 129–137. 10.1002/bmc.3249 24935248

[B33] Sun H. Chow E. C. Liu S. Du Y. Pang K. S. (2008). The Caco-2 Cell Monolayer: Usefulness and Limitations. Expert Opin. Drug Metab. Toxicol. 4 (4), 395–411. 10.1517/17425255.4.4.395 18433344

[B34] Turco L. Catone T. Caloni F. Di Consiglio E. Testai E. Stammati A. (2011). Caco-2/TC7 Cell Line Characterization for Intestinal Absorption: How Reliable Is This *In Vitro* Model for the Prediction of the Oral Dose Fraction Absorbed in Human? Toxicol. Vitro 25 (1), 13–20. 10.1016/j.tiv.2010.08.009 20732406

[B35] Wahlström B. Blennow G. (1978). A Study on the Fate of Curcumin in the Rat. Acta Pharmacol. Toxicol. (Copenh) 43 (2), 86–92. 696348 10.1111/j.1600-0773.1978.tb02240.x

[B36] Walters H. C. Craddock A. L. Fusegawa H. Willingham M. C. Dawson P. A. (2000). Expression, Transport Properties, and Chromosomal Location of Organic Anion Transporter Subtype 3. Am. J. Physiol. Gastrointest. Liver Physiol. 279 (6), G1188–G1200. 10.1152/ajpgi.2000.279.6.G1188 11093941

[B37] Wu X. W. Wang R. F. Yuan M. Xu W. Yang X. W. (2013). Dulbecco's Modified eagle's Medium and Minimum Essential Medium-Wwhich One Is More Preferred for Establishment of Caco-2 Cell Monolayer Model Used in Evaluation of Drug Absorption? Pharmazie 68 (10), 805–810. 24273884

[B38] Xiang D. Fan L. Hou X. L. Xiong W. Shi C. Y. Wang W. Q. (2018). Uptake and Transport Mechanism of Dihydromyricetin across Human Intestinal Caco-2 Cells. J. Food Sci. 83 (7), 1941–1947. 10.1111/1750-3841.14112 29969512

[B39] Xiang Q. Zhang W. Li Q. Zhao J. Feng W. Zhao T. (2020). Investigation of the Uptake and Transport of Polysaccharide from Se-Enriched Grifola Frondosa in Caco-2 Cells Model. Int. J. Biol. Macromol 158, 1330-1341. 10.1016/j.ijbiomac.2020.04.160 32339585

[B40] Yang C. Li Z. Zhang T. Liu F. Ruan J. Zhang Z. (2013). Transcellular Transport of Aconitine across Human Intestinal Caco-2 Cells. Food Chem. Toxicol. 57, 195–200. 10.1016/j.fct.2013.03.033 23562926

[B41] Ye L. Yang X. Yang Z. Gao S. Yin T. Liu W. (2013). The Role of Efflux Transporters on the Transport of Highly Toxic Aconitine, Mesaconitine, Hypaconitine, and Their Hydrolysates, as Determined in Cultured Caco-2 and Transfected MDCKII Cells. Toxicol. Lett. 216 (2-3), 86–99. 10.1016/j.toxlet.2012.11.011 23200901

[B42] Yee S. (1997). *In Vitro* permeability across Caco-2 Cells (Colonic) Can Predict *In Vivo* (Small Intestinal) Absorption in Man-Ffact or Myth. Pharm. Res. 14 (6), 763–766. 10.1023/a:1012102522787 9210194

[B43] Zha L. Y. Luo H. J. Deng H. Chu X. W. (2009). Establishment and Assessment of Caco-2 Cell *In Vitro* Absorption Model. Nan Fang Yi Ke Da Xue Xue Bao 29 (3), 548–550. 19304551

[B44] Zhang Z. D. Huang M. Z. Yang Y. J. Liu X. W. Qin Z. Li S. H. (2020). Aspirin Eugenol Ester Attenuates Paraquat-Induced Hepatotoxicity by Inhibiting Oxidative Stress. Front. Physiol. 11, 582801. 10.3389/fphys.2020.582801 33192594 PMC7642976

[B45] Zhang Z. D. Yang Y. J. Liu X. W. Qin Z. Li S. H. Li J. Y. (2021). Corrigendum to “Aspirin Eugenol Ester Ameliorates Paraquat-Induced Oxidative Damage through ROS/p38-MAPK-mediated Mitochondrial Apoptosis Pathway”. Toxicology 454, 152763. 10.1016/j.tox.2021.152763 33812688

[B46] Zhang Z. D. Yang Y. J. Liu X. W. Qin Z. Li S. H. Li J. Y. (2020). The Protective Effect of Aspirin Eugenol Ester on Paraquat-Induced Acute Liver Injury Rats. Front. Med. (Lausanne) 7, 589011. 10.3389/fmed.2020.589011 33392217 PMC7773779

[B47] Zheng Y. Benet L. Z. Okochi H. Chen X. (2015). pH Dependent but Not P-Gp Dependent Bidirectional Transport Study of S-Propranolol: The Importance of Passive Diffusion. Pharm. Res. 32 (8), 2516–2526. 10.1007/s11095-015-1640-3 25690341 PMC4891189

